# Supplementary material for: Complications of Therapeutic Plasma Exchange in Pediatric Neuroimmune Disorders
Source: Children (Basel). 2025 Oct 27;12(11):1457. doi: 10.3390/children12111457 (PMC12651235; doi:10.3390/children12111457)

Table S1: Mapping of adverse events to CTCAE grades

Observed complication	Description / Clinical manifestation	CTCAE v5.0 Term	Grouped CTCAE Grade	Comment
Hypotension	Drop in systolic BP > 20 mmHg during or after TPE	Hypotension	Mild / Moderate (Grade 1–2)	Responded to fluid substitution; no vasopressors required
Hypertension	Transient blood pressure increases during TPE	Hypertension	Mild / Moderate (Grade 1–2)	Self-limiting, no pharmacologic intervention
Nausea / Vomiting	Transient nausea and/or vomiting during TPE	Nausea / Vomiting	Mild / Moderate (Grade 1–2)	Resolved spontaneously
Headache	Headache during or after TPE	Headache	Mild / Moderate (Grade 1–2)	No escalation of analgesic therapy
Abdominal pain	Cramping or discomfort during procedure	Abdominal pain	Mild / Moderate (Grade 1–2)	Transient
Pruritus / Urticaria	Itching, localized rash	Pruritus / Rash maculo-papular	Mild / Moderate (Grade 1–2)	Mild allergic-type reactions
Paresthesia / Tingling	Tingling in hands or perioral area	Paresthesia	Mild / Moderate (Grade 1–2)	Likely citrate-related
Dyspnea	Transient shortness of breath	Dyspnea	Severe (Grade 3–4)	Self-limiting, no mechanical ventilation
Catheter malfunction (non-infectious)	Occlusion, hemolysis, or clot formation	Device occlusion / Mechanical complication of catheter	Mild / Moderate (Grade 1–2)	Required flushing or replacement
Catheter infection	Local infection, erythema, or fever	Device-related infection	Severe (Grade 3–4)	In one case progressed to sepsis

 https://doi.org/10.3390/xxxxx

Sepsis (catheter-associated)	Staphylococcus aureus bloodstream infection	Sepsis	Severe (Grade 3–4)	Required systemic antibiotics; no fatal outcome
Fever	Transient fever during or after TPE	Pyrexia	Mild / Moderate (Grade 1–2)	Often infection-related
Chills / Feeling of warmth	Chills, shivering, or heat sensations	Chills / Feeling hot	Mild / Moderate (Grade 1–2)	Transient
Other (miscellaneous)	Anxiety, agitation, elevated serum calcium, neck pain	Anxiety / Agitation / Metabolic disturbance	Mild / Moderate (Grade 1–2)	Rare, self-limiting

Figure S1: Patient inclusion flow diagram

Flow diagram illustrating patient inclusion and data availability. A total of 53 pediatric patients with neuroimmune disorders (PNID) who underwent therapeutic plasma exchange (TPE) were identified. Six patients were excluded from specific analyses due to incomplete documentation, resulting in 47 patients (365 procedures) included in the complication analysis. Data on treatment characteristics were available for 48 patients, and outcome analysis was performed in 45 patients with documented clinical status at hospital discharge.

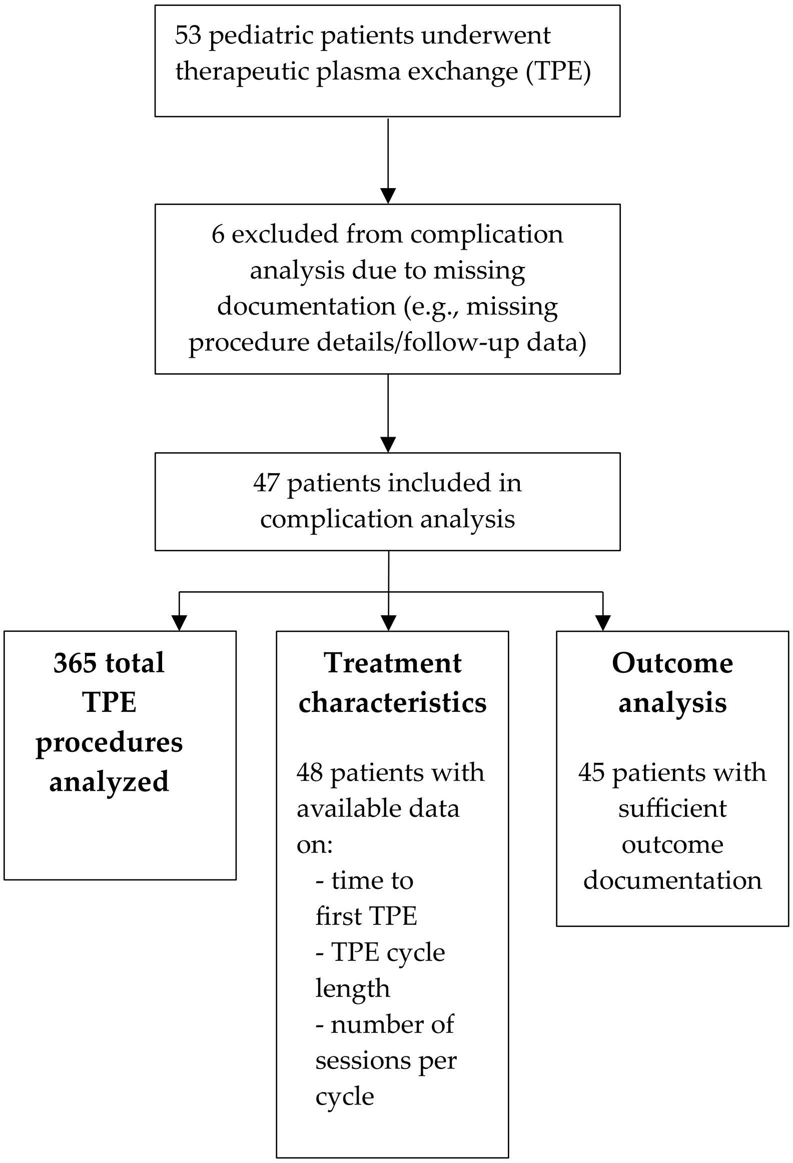

Supplement: Supplementary file 1 [file children-12-01457-s001.zip › children-3895635-supplementary.pdf]
